# The mediating effect of resilience on the relationship between competence in dementia care and empathetic practices among nurses: a cross-sectional study

**DOI:** 10.3389/fpsyg.2026.1714350

**Published:** 2026-07-06

**Authors:** Shaimaa Mohamed Amin, Mohamed H. R. Atta, Sameer A. Alkubati, Heba Ahmed Mohammed El-Fadawy, Soher Ahmed Awad Abdelaziz, Ahmed Abdelwahab Ibrahim El-Sayed, Haitham Mokhtar Mohamed Abdallah, Nesreen AbdelMonaem AbdelSataar AbouZeid, Sally Mohammed Farghaly Abdelaliem, Suebsarn Ruksakulpiwat

**Affiliations:** 1 Department of Community, Psychiatric, and Mental Health Nursing, College of Nursing, Qassim University, Buraydah, Saudi Arabia; 2 Community Health Nursing Department, Faculty of Nursing, Damanhour University, Damanhour, Egypt; 3 Nursing Services Department, Qassim University Medical City, Buraydah, Saudi Arabia; 4 Nursing Department, College of Applied Medical Sciences, Prince Sattam Bin Abdulaziz University, Wadi Addawasir, Saudi Arabia; 5 Faculty of Nursing, Alexandria University, Alexandria, Egypt; 6 Department of Medical-Surgical Nursing, College of Nursing, University of Ha’il, Ha’il, Saudi Arabia; 7 Department of Gerontological Nursing, Faculty of Nursing, Mansoura University, Mansoura, Egypt; 8 Department of Gerontological Nursing, Faculty of Nursing, Assiut University, Assiut, Egypt; 9 Faculty of Nursing, Zarqa University, Zarqa, Jordan; 10 Department of Nursing Administration, Faculty of Nursing, Alexandria University, Alexandria, Egypt; 11 Department of Medical-Surgical Nursing, College of Nursing, Jouf University, Sakaka, Saudi Arabia; 12 Critical Care and Emergency Nursing, Faculty of Nursing, Alexandria University, Alexandria, Egypt; 13 Department of Medical and Surgical Nursing, College of Nursing, Princess Nourah bint Abdulrahman University, Riyadh, Saudi Arabia; 14 Department of Nursing, Faculty of Allied Health Sciences, Kuwait University, Kuwait City, Kuwait; 15 Department of Medical Nursing, Faculty of Nursing, Mahidol University, Bangkok, Thailand

**Keywords:** cross-sectional study, dementia care, Egypt, empathetic practices, mediation, nurses, nursing competence, resilience

## Abstract

**Background:**

Dementia care requires not only clinical competence but also a high level of empathy from nursing professionals. Resilience has increasingly been recognized as a critical factor in enhancing both competence and compassionate care in demanding healthcare settings.

**Objective:**

This study aimed to examine the mediating role of resilience in the relationship between nurses’ competence in dementia care and their empathetic practices.

**Methods:**

This cross-sectional descriptive study was conducted among 200 registered nurses from ten hospitals in Sohag and Mansoura, Egypt. Participants met specific inclusion criteria, including at least one year of dementia care experience. Data were collected using the following instruments; the Connor–Davidson Resilience Scale, the Empathetic Care Scale, and the Sense of Competence in Dementia Care Staff Scale were administered. Data were analysed using descriptive statistics, Pearson correlation, and mediation analysis.

**Results:**

Significant positive correlations were found between nurses’ competence in dementia care and resilience (r = 0.350, *p* < 0.001), and between competence and empathetic practices (r = 0.249, *p* < 0.001). Resilience was also significantly correlated with empathetic practices (r = 0.378, *p* < 0.001). Mediation analysis revealed that resilience partially mediated the relationship between competence and empathetic practices (indirect effect: *β* = 0.116, BootSE = 0.036, 95% CI [0.053, 0.193]). The total effect of competence on empathetic practices was statistically significant (β = 0.249, B = 0.301, SE = 0.083, t = 3.625, *p* < 0.001), explaining 6.2% of the variance.

**Conclusion:**

Resilience serves as a key psychological mechanism through which nurses’ competence in dementia care translates into higher levels of empathetic practice. These findings underscore the importance of incorporating resilience-building interventions into nursing education and practice to enhance the quality of dementia care.

## Introduction

The increasing global aging population necessitates the availability of qualified nurses to deliver specialized care for older adults (Fedele, 2015). However, adverse working conditions in geriatric care settings have contributed significantly to both national and international nursing shortages (Christopher et al., 2015; Shaban et al., 2025a). Nurses working with older people often face low professional status and physically and emotionally demanding workloads (Schmidt et al., 2012). Dementia is characterized by a substantial decline in cognitive domains such as attention, memory, executive functioning, language, perceptual-motor abilities, and social cognition. This deterioration impairs individuals’ ability to perform everyday activities independently and often leads to behavioral changes that necessitate support with complex tasks (APA, 2013; Gale et al., 2018).

Currently, dementia arises due to multiple conditions and injuries impacting brain function, with Alzheimer’s disease accounting for most cases, approximately 60 to 70% (WHO, 2025), and projections indicate this number may increase to 135.5 million by 2050 (Cooper, 2018). The increasing prevalence of dementia and the aging global population have escalated the demand for healthcare services across various settings, including hospitals, care homes, communities, and households (Jones and Dolsten, 2024; Khalil et al., 2025).

Healthcare professionals, such as nurses, form one of the core groups of dementia care staff, each playing essential roles that demand specific competencies (Kunkle et al., 2020). Dementia care competence, broadly understood as the ability gained through hands-on experience to deliver effective care to people with dementia, has gained recognition as a critical factor in caregiving quality (Brown et al., 2023; Rivett et al., 2019). Evidence suggests that higher competence among care staff correlates with improved outcomes for persons with dementia, including lower incidences of aggression and agitation, reduced reliance on physical restraints or antipsychotic medication, and overall better quality of life and safety (Lim and Gu, 2022; Sefcik et al., 2022).

Additionally, competent staff can make more efficient use of healthcare resources, enhance caregiver support, and increase job satisfaction and staff retention (Rasmussen et al., 2023). Therefore, understanding dementia care competence and the factors that influence it is essential for improving care outcomes. Research shows that elements such as education level, frequency of training, and prior work experience positively impact care competence (Schepers et al., 2012; Traynor et al., 2011). Nurses with comprehensive knowledge of dementia and mental health conditions tend to provide better care (Furåker and Nilsson, 2009; Wang et al., 2020). Moreover, a person-centered care approach not only strengthens competence in dementia care but also correlates with higher job satisfaction (Chen et al., 2023; Coppin and Fisher, 2020).

Effective communication between nurses and elderly patients must align with the patients’ cognitive abilities (Atif et al., 2024; Hazif-Thomas and Thomas, 2015). In this context, empathy plays a crucial role in fostering meaningful nurse–patient interactions (Darzi-Ramandi et al., 2023; Van der Elst et al., 2012). As a dynamic form of communication, empathy allows nurses to respond appropriately to patients’ emotions, which has been shown to enhance recovery outcomes among the elderly (Richter and Kunzmann, 2011; Seo et al., 2016). It is considered a cornerstone of high-quality geriatric nursing care (Stone, 2012). Elderly care is a reciprocal process in which nurses collaborate with older patients to address age-related challenges, including discomfort, hygiene issues, and chronic health conditions (Pooley and Cohen, 2010; Reynolds 3rd et al., 2022). Moreover, identifying less-common nursing concerns grounded in knowledge, attitudes, and empathy is vital for delivering effective care (Johnson et al., 2018; Seo et al., 2016). Studies have shown that cognitive decline in older adults can impair empathic processing (Harm et al., 2013; Hazif-Thomas and Thomas, 2015), and that empathy has a stronger influence on elderly care practices than nurses’ attitudes or clinical experiences alone (Kieboom et al., 2023).

Teófilo et al. (2019) documented that empathy remains a fundamental ethical principle in geriatric nursing, enhancing care quality and patient satisfaction (Teófilo et al., 2019). Despite its importance, a troubling decline in empathy within healthcare environments has been reported, potentially weakening the nurse–patient relationship (Serrada-Tejeda et al., 2025). Caring for older adults, especially those with dementia, increases the burden, as these patients require more time, specialized knowledge, and present unpredictable demands (Faronbi et al., 2019; Reynolds 3rd et al., 2022). Such stressors not only compromise the well-being and work ability of nurses but can also lead to burnout and higher turnover rates (Schmidt et al., 2012). Moreover, inadequate leadership and managerial support in aged care facilities contribute to job dissatisfaction and amplify emotional strain among nurses (Nikbakht Nasrabadi et al., 2021).

Despite these persistent adversities, some nurses manage to adapt, persevere, and even thrive in such environments, suggesting the presence of resilience (Benadé et al., 2017; Koen et al., 2011). Resilience is commonly defined as the capacity to adapt successfully to adversity, encompassing both everyday difficulties and major life disruptions, with positive adjustment proportional to the intensity of the stressor (Fletcher and Sarkar, 2013). It embodies the ability to “bounce back” and move forward after hardship (Earvolino-Ramirez, 2007), drawing on both internal and external resources to navigate complex contextual and developmental challenges (Pooley and Cohen, 2010). Psychological resilience promotes the effective use of personal strengths and behaviors that buffer individuals from the detrimental impacts of stress (Fletcher and Sarkar, 2013). In nursing, demonstrating resilient behaviors can empower practitioners to cope effectively with workplace stressors and reduce the risk of burnout (Shaban et al., 2025b). Thus, fostering resilience may be a key strategy in supporting nurses in geriatric care and mitigating the broader issue of workforce shortages.

Importantly, resilience may not merely accompany empathy but actively enable it. Empathy is emotionally demanding, requiring nurses to stay present with patients’ distress without becoming overwhelmed. By supporting emotional regulation and buffering chronic stress, resilience helps preserve the emotional reserves that empathy draws upon, whereas depleted or burned-out nurses tend to withdraw and disengage (Koen et al., 2011). Resilient nurses also recover more quickly from taxing encounters, sustaining the openness needed for continued compassionate care, particularly in the high-demand context of dementia (Fletcher and Sarkar, 2013).

Despite the growing recognition of empathy as a critical component of high-quality dementia care (Digby et al., 2016), nurses continue to face significant challenges in consistently demonstrating empathetic practices in hospital settings. Prior research has highlighted the burden experienced by caregivers due to disruptions in their daily activities and physical health, emphasizing the protective role of a high sense of competence in reducing such burden (Amin et al., 2025). Additionally, educational interventions such as simulation-based training have shown promise in enhancing nurses’ communication and empathy skills, particularly in urban healthcare settings (Abdel Kader and Shaban, 2025). However, while these studies underscore the importance of competence and empathy in dementia care, there is a notable gap in the literature regarding the psychological mechanisms that may strengthen or explain this relationship. Specifically, no prior study has examined the mediating role of resilience in the relationship between nurses’ competence in dementia care and their empathetic practices. Given the emotional demands of dementia caregiving, resilience may serve as a critical buffer, enabling nurses to translate their clinical competence into compassionate, patient-centered care. Our study is the first to address this gap by exploring the mediating effect of resilience in this relationship, thereby contributing new insights into how internal psychological resources can enhance the quality of dementia care provided by nurses. Before specifying this mediating pathway, however, it is useful to consider how the individual relationships among competence, resilience, and empathetic practice have been established in prior work.

Taken together, prior evidence offers preliminary support for each of the three pairwise associations in the proposed model. Competence is linked to empathetic practice, as nurses with greater dementia-specific knowledge and confidence are better able to recognize patients’ emotional states and deliver person-centered, empathetic care (Ishii et al., 2026). Competence is also associated with resilience, since a stronger perception of competence at work has been shown to foster nurses’ resilience and adaptation to occupational demands (Gagnon-Béland et al., 2025). In turn, resilience relates to empathetic practice, as more resilient nurses sustain emotional availability and compassionate engagement while remaining less vulnerable to burnout (Uguz et al., 2025). Although these associations have each received empirical attention, they have largely been examined in isolation rather than integrated into a single framework. Together, they suggest a coherent pathway in which competence relates to empathetic practice both directly and indirectly by strengthening resilience. Accordingly, the present study proposes and tests resilience as a mediator of the relationship between nurses’ competence in dementia care and their empathetic practices.

## Subjects and methods

### Study design and setting

A cross-sectional descriptive research design was employed in this study, following the guidelines outlined in the Strengthening the Reporting of Observational Studies in Epidemiology (STROBE) Checklist. The research was conducted across ten hospitals in two distinct regions of Egypt, representing a mix of urban and rural healthcare settings. The selected hospitals include both public and private healthcare facilities, providing a wide variety of clinical environments and patient demographics. These hospitals are equipped to offer comprehensive healthcare services, including specialized dementia care units, geriatric wards, and general medical and surgical departments where nurses frequently care for patients with varying degrees of dementia and cognitive impairments.

### Sample size and study population

The inclusion criteria for this study required participants to be registered nurses currently working in the selected hospitals, with direct involvement in dementia care, such as in geriatric wards, memory clinics, or specialized dementia units. Nurses with at least one year of clinical experience in dementia care were eligible to ensure adequate exposure to dementia patients and care practices. Only those who were willing to participate and provided informed consent were included.

Exclusion criteria included nurses who did not regularly care for dementia patients, such as those working in paediatric or surgical units. Nurses with diagnosed mental health conditions or undergoing psychological treatment were excluded, as these factors could impact resilience and empathetic behaviors. Temporary, part-time, or non-clinical staff, such as those in administrative or teaching roles, were also excluded, along with those who declined to participate or did not provide informed consent.

The sample size was calculated using G*Power version 3.1.9.4 (Faul et al., 2007). Based on a power of 0.95, an alpha level of 0.05, and a moderate effect size of 0.25, a minimum of 168 participants was required. To accommodate potential dropouts, the sample size was increased to 210. However, ten nurses declined to participate, resulting in a final sample of 200 participants.

### Instruments

#### The demographic form

The demographic information of the participants was collected through a structured questionnaire that included variables such as age, sex, place of residence, family income, marital status, years of experience in gerontological care, and education level. Additionally, participants were asked about the number of patients typically cared for per shift and whether they had received previous training in dementia care.

#### Connor–Davidson Resilience Scale (10-item CD-RISC)

The Connor-Davidson Resilience Scale (CD-RISC), initially developed by Connor and Davidson in 2003, is a widely used tool for measuring resilience, the capacity to adapt to and recover from adversity (Connor and Davidson, 2003). Later, Campbell-Sills and Stein (2007) refined the scale, introducing a shorter 10-item version (CD-RISC-10) that maintains strong psychometric properties (Campbell-Sills and Stein, 2007). Each item on the CD-RISC-10 is rated on a five-point Likert scale, ranging from “Not true at all” (1) to “True nearly all the time” (5), with higher scores reflecting greater resilience. The tool demonstrates excellent internal consistency, with Cronbach’s alpha typically falling between 0.85 and 0.92, and test–retest reliability estimated at 0.87, indicating strong stability over time. Its construct validity is well-supported, with factor loads ranging from 0.50 to 0.75 and positive correlations with other resilience and psychological well-being measures, thereby establishing its convergent validity. In this study, the Arabic-translated version of the CD-RISC-10 demonstrated outstanding reliability, with a Cronbach’s alpha of 0.90.

#### Empathetic Care Scale

The Empathetic Care Scale, developed by Lamberton et al. (2015), is a self-report tool designed to assess the level of empathetic care provided by healthcare professionals, particularly in gerontological settings. The scale consists of 10 items divided into three subscales: extra-role behavior (4 items), emotional support (3 items), and relational richness (3 items). Each item is rated on a 7-point Likert scale, ranging from Strongly Disagree (1) to Strongly Agree (7). A higher total score reflects a greater level of empathetic care provided by the healthcare professional. Lamberton et al. (2015) demonstrated that the scale has strong validity and reliability. The construct validity of the scale was supported through Exploratory Factor Analysis (EFA), which revealed a three-factor structure: emotional resonance, cognitive understanding, and behavioral responsiveness, which effectively represents the concept of empathetic care. Confirmatory Factor Analysis (CFA) further validated this structure, with good fit indices, including a Comparative Fit Index (CFI) of 0.96 and a Root Mean Square Error of Approximation (RMSEA) of 0.05, confirming the theoretical model. In terms of reliability, the scale showed excellent internal consistency, with a Cronbach’s alpha of 0.92, indicating that the items reliably measure the same construct. The scale also demonstrated strong test–retest reliability, with a correlation of 0.85, ensuring stability over time. In the present study, the scale’s Cronbach’s alpha was 0.86, indicating good internal consistency.

#### The Sense of Competence in Dementia Care Staff Scale

The Sense of Competence in Dementia Care Staff Scale, developed by Bian et al. (2023), is designed to assess the perceived competence of dementia care staff. It comprises 17 items across four dimensions: Building Relationships (4 items), Sustaining Personhood (4 items), Professionalism (5 items), and Care Challenges (4 items). Each item is rated on a four-point Likert scale, ranging from not at all (1) to very much (4). A higher total score indicates a greater perceived competence among dementia care staff. According to Bian et al. (2023), the scale demonstrated strong internal consistency with a Cronbach’s alpha of 0.92. Exploratory Factor Analysis (EFA) confirmed a clear factor structure, validating the scale’s construct. Confirmatory Factor Analysis (CFA) further supported this structure, with a Comparative Fit Index (CFI) of 0.95 and a Root Mean Square Error of Approximation (RMSEA) of 0.06, showing a good model fit. In our study, the Cronbach’s alpha was 0.89, reflecting good internal consistency.

### Study procedures

#### Tool preparation and pilot study

The research tools were translated into Arabic by two independent bilingual specialists proficient in both English and Arabic, having expertise in healthcare research and academic translation. Initially, the translation from English to Arabic was conducted separately by the two translators to ensure both linguistic precision and conceptual equivalence. The translated drafts were subsequently compared and synthesized into a unified preliminary Arabic version through discussions and agreements among the research team and translators.

To validate the translation’s accuracy, a backward translation into English was performed independently by two additional bilingual translators who were unaware of the original English versions of the tools. The back-translated drafts were analyzed against the original tools to pinpoint any discrepancies or conceptual variations. Any inconsistencies were examined and resolved through dialogue among the translators and the research team until a consensus was reached.

Face validity was then evaluated by an expert panel to ensure that the translated tools appropriately represented the intended constructs within the Arabic cultural context. Feedback from prospective participants was also gathered to assess the clarity, relevance, and cultural suitability of the questionnaire items. Reliability was measured using Cronbach’s alpha to evaluate internal consistency. A pilot study was subsequently carried out with 20 participants to further assess the clarity, relevance, and reliability of the tools and study procedures. Participants in the pilot study were excluded from the final sample. The pilot results indicated that the tools were clear and reliable, necessitating no further amendments.

### Data collection

The data collection process began with a brief orientation session in which participants were informed about the study objectives, procedures, and the voluntary nature of participation. Any questions or concerns raised by participants were addressed to ensure clarity and understanding, and confidentiality measures were clearly explained to build trust and encourage honest responses.

Data were collected between February 2025 and May 2025 by trained researchers, who were members of the research team and had received prior training on the study protocol, ethical conduct, and standardized questionnaire administration in accordance with Good Clinical Practice guidelines. Written informed consent was obtained from all participants before enrollment.

To ensure anonymity, participants were instructed not to include any personal identifiers on the questionnaires. Participation remained entirely voluntary, and respondents were informed of their right to withdraw from the study at any time without consequences. Data were collected through interviews conducted in the outpatient clinic waiting areas, with each interview lasting approximately 15–20 min.

### Statistical analysis

The data was analysed using IBM SPSS Statistics, Version 27 (IBM Corp., Armonk, NY, USA). The categorical variables were summed together using descriptive statistics like frequencies and percentages. Pearson’s correlation coefficient was calculated to examine the link between the variables in question. Hayes’ PROCESS Macro for SPSS, version 4.2, Model 4, was used to assess the mediating effect of resilience on the relationship between competence in dementia care and empathetic practices. The mediation analysis was conducted using 5,000 bootstrap samples and 95% bias-corrected confidence intervals.

## Results

### Sociodemographic characteristics of study participants

As described in Table 1, more than half of the study participants (51.0%) were aged over 30 years and living in urban areas. Most of the study participants were female (80%), married (65.0%), had a bachelor’s degree (58.5%), had an insufficient income (55.5%), and had experience of 10 years or less (62.0%).

**Table 1:** Sociodemographic characteristics of study participants (*n* = 200).

Variables	Categories	*n*	%
Age	33.61 ± 7.74		
	≤30	98	49.0
	>30	102	51.0
Sex			
	Male	40	20.0
	Female	160	80.0
Residence			
	Rural	98	49.0
	Urban	102	51.0
Income			
	Not enough	111	55.5
	Enough	64	32.0
	Enough and save	25	12.5
Marital status			
	Single	37	18.5
	Married	130	65.0
	Divorced/Widowed	33	16.5
Level of education			
	Diploma	42	21.0
	Bachelor	117	58.5
	Master	41	20.5
Experience	11.05 ± 10.74		
	≤10	124	62.0
	>10	76	38.0

### Correlation between study variables

There was a significant positive correlation between nurses’ competence in dementia care and resilience (r = 0.350, *p* < 0.001), as well as between competence and empathetic practice (r = 0.249, *p* < 0.001). Additionally, resilience was significantly correlated with empathetic practice (r = 0.378, *p* < 0.001) (Table 2).

**Table 2:** Correlation between study variables.

Variables	Competence	Resilience	Empathetic practices
Competence	1		
Resilience	0.350**	1	
Empathetic practices	0.249**	0.378**	1

**Correlation is significant at *p*-value < 0.01 level (2-tailed).

### The mediating effect of resilience on the relationship between competence and empathetic practices

**Figure 1:**
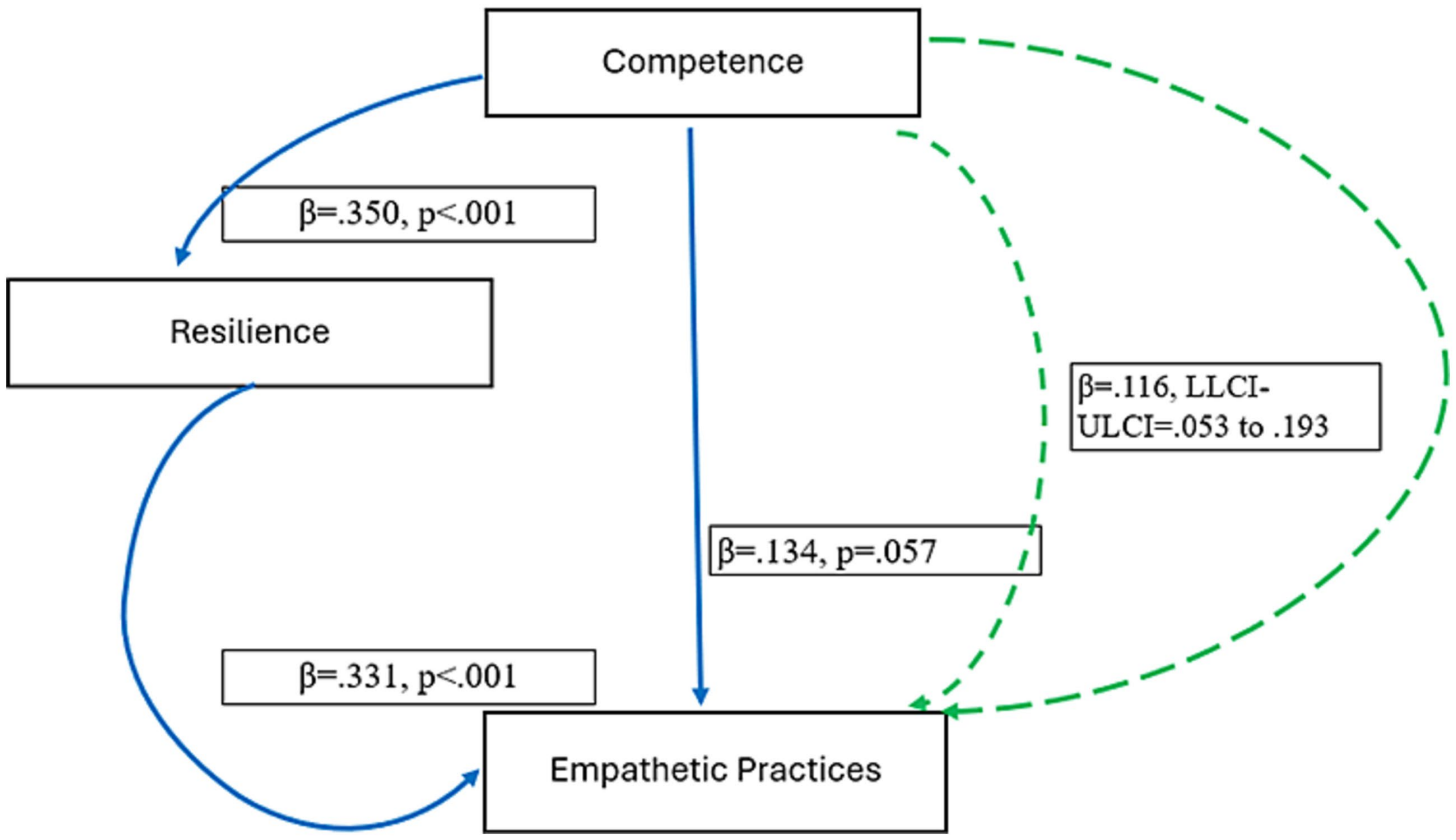
The mediating effect of resilience on the relationship between competence and empathetic practices.

The mediation model was tested using the PROCESS macro for SPSS (Model 4), with empathetic practices as the dependent variable, resilience as the mediating variable, and competence as the independent variable. Figure 1 and Table 3 present the direct, indirect, and total effects of competence on empathetic practices, with resilience examined as a mediator. In the mediator model, competence had a significant positive effect on resilience (*β* = 0.350, B = 0.395, SE = 0.075, t = 5.258, *p* < 0.001), explaining 12.3% of the variance.

**Table 3:** Direct, indirect, and total effects of competence on empathetic practices.

Models	B	β	*SE*	t	95% CI	*p*-value
MV model						
Competence → Resilience	0.395	0.350	0.075	5.258	0.247 to 0.543	<0.001
Model summary R^2^ = 0.123, *F* = 27.642, *p* < 0.001						
DV model						
Resilience → Empathetic practices	0.354	0.331	0.075	4.742	0.207 to 0.501	<0.001
Competence → Empathetic practices	0.161	0.134	0.084	1.915	−0.005 to 0.327	0.057
Model summary R^2^ = 0.158, *F* = 18.524, *p* < 0.001						
Total effect						
Competence → Empathetic practices	0.301	0.249	0.083	3.625	0.137 to 0.465	<0.001
Model summary R^2^ = 0.062, *F* = 13.138, *p* < 0.001						

Indirect effect	β	BootSE	BootLLCI	BootULCI
Competence → Resilience → Empathetic practices	0.116	0.036	0.053	0.193

B = unstandardized coefficient; β = standardized coefficient; SE = standard error; t = t-value; CI = confidence interval; LLCI = lower limit of confidence interval; ULCI = upper limit of confidence interval; p = probability value; R^2^ = coefficient of determination; F = F-statistic; MV = mediator variable model; DV = dependent variable model; BootSE = bootstrap standard error.

In the direct effects model, resilience was a significant positive predictor of empathetic practices (β = 0.331, B = 0.354, SE = 0.075, t = 4.742, *p* < 0.001). However, the direct effect of competence on empathetic practices was not statistically significant (β = 0.134, B = 0.161, SE = 0.084, t = 1.915, *p* = 0.057). The model accounted for 15.8% of the variance in empathetic practices.

The total effect of competence on empathetic practices was statistically significant (β = 0.249, B = 0.301, SE = 0.083, t = 3.625, *p* < 0.001), explaining 6.2% of the variance. Bootstrapping analysis revealed a significant indirect effect of competence on empathetic practices through resilience (β = 0.116, BootSE = 0.036, 95% CI [0.053, 0.193]), as the confidence interval did not include zero.

## Discussion

This study examined the relationships among nurses’ competence in dementia care, resilience, and empathetic practice, and tested resilience as a mediator. Three main findings emerged: competence was positively associated with empathetic practice, competence was positively associated with resilience, and resilience was positively associated with empathetic practice. Critically, resilience partially mediated the competence–empathy relationship. This nuanced understanding adds a new dimension to the literature by highlighting resilience not just as a coping strategy, but as a psychological bridge that enables competent nurses to deliver emotionally resonant care.

The significant direct relationship between competence and empathetic practice confirms the assertion that clinical knowledge is foundational for effective interpersonal engagement in dementia care. Nurses with higher perceived competence were more likely to engage in empathetic behaviors, echoing earlier studies by Wang et al. (2020) and Furåker and Nilsson (2009), which found that comprehensive knowledge of dementia and mental health supports a better understanding of patients’ needs, promotes accurate interpretation of behavioral cues, and fosters person-cantered responses. These findings are in line with the broader framework of person-centered care, which emphasizes that clinical skill must be paired with a humanistic approach to achieve quality care outcomes (Chen et al., 2023; Shoukr et al., 2025).

However, other scholars challenge the assumption that competence alone ensures empathy. Van der Elst et al. (2012) noted that while technical skills are essential, they are insufficient without emotional presence and reflective capacity. Their findings suggest that systemic constraints such as understaffing, time pressures, and inadequate leadership can blunt the emotional expression of even the most competent nurses. Similarly, Tural et al. (2015) reported variations in empathy levels among highly trained oncology nurses, implying that emotional attune does not always accompany technical expertise. These contrasting findings underscore that while competence provides a structural foundation for care, the emotional climate of caregiving is influenced by more fluid psychological factors.

The positive association between competence and resilience is central to the mediation framework and warrants explicit attention. Nurses who perceived themselves as more competent also reported greater resilience, suggesting that a secure sense of one’s own clinical ability functions as a psychological resource that supports adaptive coping (Cabrera-Aguilar et al., 2023). This is consistent with Gagnon-Béland et al. (2025), who found that a stronger perception of competence at work fosters nurses’ resilience and adaptation to occupational demands. A plausible interpretation is that competent nurses experience greater self-efficacy and control in challenging caregiving situations, which buffers stress and strengthens their capacity to recover from adversity. In the context of dementia care, where unpredictable behaviors and emotional strain are routine, competence may therefore protect nurses from depletion and lay the groundwork for sustained empathetic engagement.

In this context, the present study’s identification of resilience as a mediator brings critical depth to the understanding of how competence translates into empathy. Resilience, as conceptualized by Fletcher and Sarkar (2013), is not merely a buffer against stress but a proactive capacity that enables individuals to recover from adversity and maintain interpersonal connections. The findings align with this framework: nurses who reported greater resilience also demonstrated more empathetic behaviors, indicating that resilience helps preserve emotional availability even in high-stress dementia care environments. This supports the argument made by Koen et al. (2011), who found that resilient nurses exhibited better emotional regulation, sustained motivation, and reduced vulnerability to burnout, thereby maintaining relational effectiveness with patients.

Moreover, the partial mediation observed in the current model suggests that resilience acts in concert with, rather than independently of, other variables in shaping empathetic practices. Specifically, resilience accounted for part, but not all, of the association between competence and empathetic practice. Although the direct effect of competence was attenuated and no longer statistically significant once resilience was entered into the model, the total effect remained significant, indicating that resilience explains only part of this relationship and that additional mechanisms are likely involved. The psychological flexibility afforded by resilience likely supports nurses in navigating the moral complexities and emotional tolls of dementia care, enabling them to uphold compassionate care standards despite adversity. Amin et al. (2025a) further advanced this view by proposing that moral resilience enhances ethical decision-making and protects against emotional fatigue capacities that are vital in geriatric and dementia-focused nursing (Amin et al., 2025a). Importantly, because the present design was cross-sectional, this mediation should be interpreted as associational rather than causal, as the data cannot establish the temporal ordering required to confirm a true mediating pathway.

While these insights affirm the centrality of resilience, they also open space for further exploration of other mediators. Emotional intelligence, for instance, may play a complementary or synergistic role in translating competence into empathetic engagement. Serrada-Tejeda et al. (2025) and Richter and Kunzmann (2011) found that emotional intelligence significantly predicts empathy in nurses, suggesting that resilience alone may not fully account for the variability in empathetic behavior. Furthermore, organizational support and leadership quality could function as external moderators, as Digby et al. (2016) observed that workplace constraints such as rigid hierarchies and limited autonomy can inhibit even resilient nurses from fully exercising empathetic care.

The demographic profile of the study participants adds another layer of interpretive richness. Clibbens et al. (2019) and Benadé et al. (2017) observed that the sample predominantly consisted of female nurses with fewer than ten years of dementia care experience, a pattern also reflected in the present study. These studies found that younger, predominantly female caregivers often enter the profession with high intrinsic motivation and empathy but may lack the coping strategies required to sustain these emotional investments under pressure. The present study’s finding that resilience mediates the link between competence and empathy in such populations is therefore especially pertinent; it suggests that building resilience early in nurses’ careers could have long-term benefits for patient care quality.

At the same time, gender-specific dynamics must be acknowledged. Research by Amin et al. (2025b) and Serrada-Tejeda et al. (2025) indicated that female nurses often score higher on measures of empathy but may also experience greater emotional strain. This duality reinforces the value of resilience as both a protective and enabling factor. In a healthcare culture where emotional labor is often feminized and undervalued, fostering resilience could be a key strategy to preserve botAminh empathy and well-being among female nursing staff.

Socioeconomic conditions may also shape the relationship between resilience and empathetic practice. Over half of the participants in this study reported inadequate income and lived in urban areas, a finding that aligns with concerns raised by Isaksson et al. (2008), who linked financial stress and environmental adversity to diminished emotional responsiveness among caregivers. This contextual variable further affirms that resilience should not be treated as a purely internal trait, but as a construction shaped by both individual resources and structural conditions. A nurse’s capacity to be resilient and empathetic may be constrained or facilitated by their broader life circumstances, making institutional support mechanisms more crucial.

In terms of practical application, these findings strongly advocate for the integration of resilience training within nursing education and professional development. Traditional training approaches have prioritized technical competence through clinical simulations and theoretical modules. However, the current evidence suggests that this is not sufficient for developing holistic care capacity. Emotional resilience must be cultivated alongside technical skills, using methods such as reflective journaling, peer support groups, mindfulness exercises, and narrative medicine. Jackson et al. (2007) emphasized that resilience-building practices can help nurses develop a stronger sense of agency and meaning in their work, which in turn promotes empathetic behavior. Simulation-based learning, such as that described by Abdel Kader and Shaban (2025), is also valuable, particularly when accompanied by structured emotional debriefings and opportunities for self-reflection. Mentorship and team-based learning environments further support the development of both resilience and empathy. Zimmerman et al. (2005) noted that shared experiences and interprofessional dialogue promote emotional understanding and reduce burnout, allowing nurses to better integrate competence with compassion. These interpersonal dynamics are vital in dementia care, where emotional demands are high and patient communication is often nuanced and complex. Regular supervision, ethical case discussions, and opportunities for role-modeling empathetic behavior can serve as scaffolding for less experienced nurses.

Beyond the institutional setting, this study contributes significantly to the global dialogue on dementia care by situating its findings in a non-Western context. The rigorous translation and validation of assessment instruments into Arabic enhances the cultural sensitivity and generalizability of the results. As dementia becomes a global health priority, culturally grounded models of care are urgently needed (Brown et al., 2023; WHO, 2025). Khalil et al. (2025) noted that community attitudes toward aging and cognitive decline vary widely, influencing both professional caregiving practices and family support structures. By validating a resilience-based model of empathetic nursing in Egypt, this study offers a culturally adaptive framework that can inform practice and policy in other Middle Eastern and low- to middle-income contexts.

The intersection of competence, resilience, and empathy presents a multidimensional framework for dementia care, in which each component enhances the others. Competence provides cognitive and procedural tools for effective caregiving. Resilience allows for the emotional sustainability of care, enabling nurses to withstand stress without emotional withdrawal. Empathy acts as the relational glue that binds technical knowledge to patient experience. The synergy among these elements affirms the holistic nature of dementia care and offers practical guidance for the training and support of nurses in this field.

### Strengths and limitations

A key strength of this study lies in its robust methodological framework and use of validated, culturally adapted instruments with strong psychometric properties. The inclusion of a diverse sample of nurses from both public and private hospitals across two governorates enhanced the generalizability of findings across various clinical settings. The rigorous translation and validation process ensured the reliability and cultural relevance of the tools used, such as the Arabic versions of the CD-RISC, the Empathetic Care Scale, and the Sense of Competence in Dementia Care Staff Scale. Furthermore, adherence to the STROBE guidelines and ethical principles, along with a well-structured pilot study, strengthened the scientific rigor and credibility of the research process.

Despite its strengths, the study has several limitations that warrant consideration. The cross-sectional design restricts the ability to establish causal relationships between competence, resilience, and empathetic practices. In particular, it precludes determining the temporal sequence among the variables, so the observed mediation cannot be interpreted as evidence of causal or temporal mediation; longitudinal designs are needed to confirm the proposed pathway. The reliance on self-reported measures may introduce response bias or social desirability effects, potentially inflating reported levels of competence or empathy. Additionally, the exclusion of nurses with mental health conditions may limit the applicability of findings to all nursing populations, as these individuals may face unique challenges related to resilience. Finally, although the sample was relatively diverse, it was limited to two governorates, which may affect the broader applicability of the results across Egypt or other cultural contexts.

### Implications

The findings of this study highlight the importance of embedding resilience-building strategies within nursing education to enhance both clinical competence and empathetic care, particularly in dementia care settings. Nursing curricula should integrate resilience training alongside dementia-specific content to better prepare nurses for the emotional and cognitive demands of working with this vulnerable population. Simulation-based learning, reflective exercises, and mentorship programs can cultivate both the technical and emotional capacities needed for compassionate dementia care. In practice settings, nurse leaders and administrators should prioritize the development of staff resilience through ongoing professional development, psychological support systems, and team-based care models that value emotional well-being as much as clinical proficiency.

From a research perspective, this study opens avenues for further investigation into the mechanisms by which resilience influences nursing outcomes. Future studies should explore the long-term effects of resilience-enhancing interventions on empathy and care quality, as well as consider other psychological factors—such as emotional intelligence or burnout—that may mediate or moderate this relationship. Additionally, cross-cultural studies are warranted to understand how different healthcare contexts shape the role of resilience in dementia care. These implications underscore the necessity of a holistic approach to nurse development—one that balances competence with emotional resilience to ensure high-quality, empathetic care for individuals living with dementia.

## Conclusion

The present study highlights the pivotal role of resilience as a psychological mechanism linking nurses’ competence in dementia care to their empathetic practices. The findings demonstrated that higher competence in dementia care was significantly associated with both greater resilience and more empathetic behavior among nurses. Notably, resilience served as a significant mediator, suggesting that competence alone is not sufficient to foster empathetic practice rather, the ability to cope adaptively with the emotional and professional demands of dementia care is equally critical. These results underscore the importance of integrating resilience-building strategies into training programs aimed at enhancing dementia care competencies. Strengthening nurses’ resilience may not only improve their capacity to manage complex caregiving situations but also enhance the quality of compassionate care delivered to individuals with dementia. Future research and nursing education should prioritize resilience as a key component in developing both clinical and interpersonal competencies among nurses working in geriatric and dementia-focused settings.

## Data Availability

The original contributions presented in the study are included in the article/supplementary material, further inquiries can be directed to the corresponding author.
